# Multicentric study on surgical information and early safety and performance results with the Bonebridge BCI 602: an active transcutaneous bone conduction hearing implant

**DOI:** 10.1007/s00405-022-07792-y

**Published:** 2023-01-10

**Authors:** Georg Sprinzl, Joseph Toner, Assen Koitschev, Nadine Berger, Thomas Keintzel, Thomas Rasse, Wolf-Dieter Baumgartner, Clemens Honeder, Astrid Magele, Stefan Plontke, Gerrit Götze, Joachim Schmutzhard, Philipp Zelger, Stephanie Corkill, Thomas Lenarz, Rolf Salcher

**Affiliations:** 1 Hals-Nasen-Ohren-Abteilung, Karl Landsteiner Privatuniversität für Gesundheitswissenschaften und Karl-Landsteiner Institut für Implantierbare Hörsysteme, Universitätsklinikum St. Pölten, Dunant-Platz 1, 3100 St. Pölten, Austria; 2 Regional Auditory Implant Centre, Beech Hall Centre, Belfast, Northern Ireland, UK; 3 Klinik für HNO-Krankheiten, Plastische Operationen, Klinikum Stuttgart, Olgahospital, Stuttgart, Germany; 4 Abteilung für Hals-, Nasen-, Ohrenkrankheiten, Klinikum Wels-Grieskirchen, Wels, Austria; 5 Allgemeines Krankenhaus der Stadt Wien, Universitätsklinik für Hals-, Nasen- und Ohrenkrankheiten, Vienna, Austria; 6 Department of Otorhinolaryngology, Head and Neck Surgery, Martin Luther University Halle-Wittenberg, Halle (Saale), Germany; 7 Universitätsklinik für Hals-, Nasen- und Ohrenheilkunde Innsbruck, Medizinische Universität Innsbruck, Innsbruck, Austria; 8 Universitätsklinik für Hör-, Stimm- und Sprachstörungen, Medizinische Universität Innsbruck, Innsbruck, Austria; 9 Medizinische Hochschule Hannover, Klinik und Poliklinik für HNO-Heilkunde, Hannover, Germany

**Keywords:** Bone conduction, Conductive hearing loss, Mixed hearing loss, Single-sided sensorineural deafness, Bonebridge, Bone conduction implant, Transcutaneous hearing implant

## Abstract

**Aim:**

This European multicentric study aimed to prove safety and performance of the Bonebridge BCI 602 in children and adults suffering from either conductive hearing loss (CHL), mixed hearing loss (MHL), or single-sided sensorineural deafness (SSD).

**Methods:**

33 patients (13 adults and 10 children with either CHL or MHL and 10 patients with SSD) in three study groups were included. Patients were their own controls (single-subject repeated measures), comparing the unaided or pre-operative to the 3-month post-operative outcomes. Performance was evaluated by sound field thresholds (SF), word recognition scores (WRS) and/or speech reception thresholds in quiet (SRT) and in noise (SNR). Safety was demonstrated with a device-specific surgical questionnaire, adverse event reporting and stable pure-tone measurements.

**Results:**

The Bonebridge BCI 602 significantly improved SF thresholds (+ 25.5 dB CHL/MHL/SSD), speech intelligibility in WRS (+ 68.0% CHL/MHL) and SRT in quiet (− 16.5 dB C/MHL) and in noise (− 3.51 dB SNR SSD). Air conduction (AC) and bone conduction (BC) thresholds remained stable over time. All adverse events were resolved, with none unanticipated. Mean audio processor wearing times in hours [h] per day for the CHL/MHL group were ~ 13 h for adults, ~ 11 h for paediatrics and ~ 6 h for the SSD group. The average surgical length was 57 min for the CHL/MHL group and 42 min for the SSD group. The versatility of the BCI 602 (reduced drilling depth and ability to bend the transition for optimal placement) allows for treatment of normal, pre-operated and malformed anatomies. All audiological endpoints were reached.

**Conclusions:**

The Bonebridge BCI 602 significantly improved hearing thresholds and speech understanding. Since implant placement follows the patient’s anatomy instead of the shape of the device and the duration of surgery is shorter than with its predecessor, implantation is easier with the BCI 602. Performance and safety were proven for adults and children as well as for the CHL/MHL and SSD indications 3 months post-operatively.

## Introduction

The Bonebridge (BB) system, the first active (direct-drive) transcutaneous Bone Conduction Implant (BCI), augments hearing by providing acoustic input to the inner ear via bone conduction and has been implanted around the world for more than 10 years.

Bone Conduction Implants offer a valuable treatment option for people who cannot wear conventional acoustic hearing aids for medical reasons, or who are unsuccessful acoustic hearing aid users [1, 2]. In addition to hearing glasses and bone conduction headbands, there are also implantable bone conduction systems. BCIs are subdivided into passive percutaneous (Baha Connect, Ponto), passive transcutaneous (Baha Attract, Sophono,) or active transcutaneous (Bonebridge, OSIA) bone conduction devices [3].

The advantage of the Bonebridge compared to passive bone conduction systems is that it bypasses attenuation through the skin [4]. Compared to percutaneous bone conduction systems, the transcutaneous implants leave the skin intact and thus reduce the risk of implant loss, infection and the need for constant wound care [5].

**Fig. 1:**
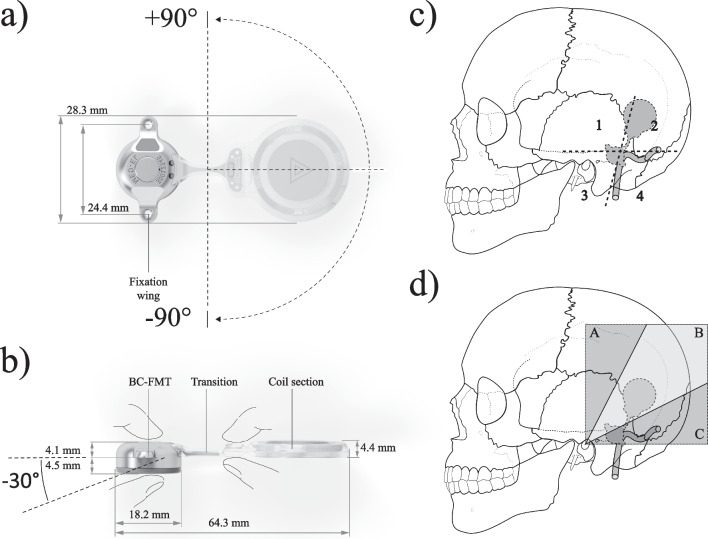
Schematics for placement evaluation: **a** BCI 602 dimensions and horizontal bending angles; **b** BCI 602 dimensions and vertical bending angles. Index fingers and thumbs should be placed at the FMT and the positioning aid, respectively; **c** BC-FMT (Bone Conduction—Floating Mass Transducer) placement areas (1, 2, 3, 4) temporal bone; **d** coil section placement areas (A, B, C)

The Bonebridge is intended to treat patients 5 years and older suffering from either conductive (CHL) or mixed hearing loss (MHL) or single-sided sensorineural deafness (SSD). The latest generation of the Bonebridge, the BCI 602, comes with self-drilling screws and an optimized design. These new features mean that the implant is suitable for implantation in a wider range of anatomical conditions due to a reduced drill depth [6, 7]. Fewer surgical steps also simplify and shorten the surgical procedure. The dimensions of the BCI 602 (MRI-conditional at 1.5 T) and its predecessor, the BCI 601, were described in a monocentric study by Cywka et al. [8] (see also Fig. 1a + b).

Previous prospective multicentric studies on the predecessor BCI 601 have shown significant improvements in terms of aided sound field (SF) thresholds, word recognition scores (WRS), speech reception thresholds in quiet and in noise (SRT) and patient device satisfaction [9–12]. Safety of the device was established with stable residual hearing and low complication rates. Here, we present surgical information and early safety, and performance results up to 3-month post-implantation from a long-term European multicentric study. These results confirm the short term safety and performance of the Bonebridge BCI 602 for its indications.

## Methods

### Ethical considerations

The study was conducted in agreement with the Declaration of Helsinki 2013 and was approved by the relevant ethics committees (Stuttgart (GER) ref.# 67634_2019BO1; Wels (A) 1168_2019; Wien (A) ref.# 1877_2019; St. Pölten (A) ref.# GS1-EK-3_159-2019: Halle (Saale) (GER) ref.# 2019–126; Innsbruck (A) ref.# 1187/2019; Belfast (UK/NIR) ref.# ORECNI 20-NI-0020; Hannover (GER) ref.# 8640_BO_S_2019). The study is registered at ClinicalTrials.gov under NCT04427033.

### Study design

The study was observatory, non-interventional, systematic, longitudinal, ambidirectional (retrospective and prospective), multicentric and multilingual, open-label, single-patient, using repeated measures. This study was designed to closely follow and observe the actual routine clinical practice of all included sites. The study comprised three groups, CHL/MHL Adult (18 years and older), CHL/MHL Paediatric (5–17 years) and SSD (5 years and older). The interim analysis per group was planned when 3-month data for the sample size of at least 6 patients were available.

### Patients

Subjects 5 years of age and older in the indication range of the BCI 602 for CHL/MHL and SSD were included. Subjects with unstable hearing loss (fluctuation of > 15 dB HL over a 2-year period), with physical, psychological and emotional diseases or disorders or simultaneous participation in another clinical trial that would interfere with the ability to perform on test procedures were not eligible for enrolment.

### Endpoints and statistical analysis

The clinical trials for the BCI 601 in adults [9] and children [10] showed significant improvements of PTA_4_ (PTA_4_ = 0.5, 1, 2 and 4 kHz) sound field thresholds. For the primary endpoint a mean improvement of ≥ 10 dB in PTA_4_ sound field thresholds was considered significant. Therefore, for this study we calculated a sample size of 5 subjects with a power of 95% and an alpha-level of 0.05 for a two-sided paired sample *t* test. To allow for possible dropouts (approximately 20%), the minimum sample size was set to 6 subjects per group.

For the secondary endpoints mean differences of > 10 dB in BC PTA_4_ and mean improvements of ≥ 15% in WRS, ≥ 15 dB SPL in SRT and ≥ 1.0 dB SNR in SRT in noise were considered significant [9, 10, 13–16]. For speech reception in noise two decimal places are reported as the improvement endpoint is 1.0 dB SNR, for all others one decimal place is clinically relevant.

Descriptive statistics were used to report patient demographics (e.g., age and gender) and baseline characteristics (e.g., aetiologies, medical history). Mean, standard deviation (SD), and/or median with range (minimum and maximum values) were used to describe quantitative data; absolute and relative frequencies were used to present qualitative data. To choose whether a parametric (two-sided *t* test; *t*) or a non-parametric test (2 sided Wilcoxon signed-rank test; *z*) should be applied, the Kolmogorov–Smirnov test and a graphical examination were conducted to check for distribution. The *t* or *z* value indicates the test used. Statistical significance was set to *p* ≤ 0.05. The corresponding confidence level is 95%. The analysis was carried out on the ITT population.

IBM SPSS Statistics 24 (IBM, Armonk, New York) was used for the analyses. Graphs were created in GraphPad Prism 6–7 (GraphPad Software, Inc.).

### Audiometric testing

Audiometric tests were scheduled before surgery and 3 months after surgery. Pure tone audiometry was performed for both ears using insert earphones, headphones, or a calibrated BC vibrator, as appropriate, on each ear individually. Thresholds were routinely measured at 0.5, 1.0, 2.0, 3.0 and 4.0 kHz for BC and additionally at 6.0 and 8.0 kHz for AC. The contra-lateral ear was plugged and covered, and masking noise was applied as needed. In SSD patients the BC thresholds of the normal-hearing ear (NH) were analysed.

For tests in the sound field, the speaker was at least 1 m from and﻿ at level with the centre of the patient’s head. Sound field (SF) thresholds and speech tests in quiet were conducted with signals presented at 0° azimuth (S0). The contra-lateral ear was plugged and covered, and/or masking noise was applied as needed in CHL/MHL patients. Aided testing was conducted with the patient wearing the audio processor (AP).

For CHL/MHL subjects, WRS were routinely measured with age-appropriate monosyllabic (MS) word lists (e.g., Freiburger, Göttinger or Mainzer) at 65 dB SPL and the percent correct score was recorded. As all lists are used to test the percent correct at the same fixed level, improvements can be pooled.

The speech reception threshold (SRT) in quiet and in noise was tested using the international matrix test (IMT) distributed by HörTech GmbH (Oldenburg), which has excellent comparability across languages [17]. The SRT is defined as the level of 50% intelligibility of spondees or comparable speech material in dB SPL. The speech presentation level was started at 65 dB SPL and varied until a patient understood approximately 50% of the presented speech. In SRT in noise, the noise level was fixed at 65 dB SPL. The difference between the level in dB at 50% speech reception and 65 dB noise level was presented as the signal-to-noise-ratio (SNR). The IMT in noise was conducted for CHL/MHL with speech (S) and noise (N) at 0° azimuth (S0°N0°) and for SSD patients with speech from the SSD ear and noise from the normal hearing ear NH (S_SSD_ N_NH_) as well as with speech from the front and noise from the NH (S0°N_NH_).

## Results

### Demographics

A total of 33 patients with an average age of 31 years (range 5–69) were analysed. All 23 patients suffering from CHL/MHL had a PTA_4_ air bone gap greater than or equal to 15 dB.

The adult CHL/MHL group encompassed eight female, five male, eight right-side and five left-side implanted patients (see Table 1), with an average age of 43 years. Seven adult patients had BC thresholds worse or equal to 20 dB HL in one or more frequency, e.g., a mixed hearing loss. Most of the patients’ hearing loss was attributed to cholesteatoma (6x), followed by congenital reasons (5x, e.g., malformation, dysplasia etc.), mastoidectomy (1x) and radical cavity (1x) (see Table 1). Two patients had been treated with a BCI 601 on the study ear 7 years earlier. Both devices had been placed in a radical cavity that was found to be infected during explantation. Examination of the explanted BCI 601 devices did not reveal any device defects or problems that could have existed, while they were implanted. In both cases the BCI 602 was then implanted above the temporal line with no connection to or away from the radical cavity.

**Table 1:** Demographic: M = male; F = female; HL = Hearing Loss; R = right; L = left; Preoperative AC and BC PTA_4_ are reported for the implant ear for CHL/MHL and the normal hearing (NH) ear of the SSD patients

ID	SEX	AGE	HL Type	AC PTA_4_	BC PTA_4_	BB Side	Aetiology/disease/previous surgeries
				(SSD NH)	(SSD NH)		
1	M	44	MHL	58.8	18.8	R	Cholesteatoma
2	M	38	MHL	36.3	11.3	L	Cholesteatoma, stenosis of the EAC
3	F	27	CHL	47.5	5	L	Cholesteatoma, Radical Cavity
4	F	63	CHL	67.5	5	R	Cholesteatoma, Mastoidrevision, 3 × Cholesteatoma removal/resection, BCI 601 explantation (implanted for 7 years) on the right side occurred 1 year before BCI 602 implantation
5	F	47	CHL	52.5	10	R	Malformation, Radical Cavity, EAC Plastic, 5 × Tympanoplasty right, Incision Scar, lesion left in high frequencies; direct exchange from a BCI 601 (implanted for 7 years) to a BCI 602
6	F	19	CHL	46.3	6.3	R	Malformation, Atresia EAC
7	M	19	CHL	40	6.3	R	EAC Atresia, Dysplasia, Sophono explantation right same year
8	F	57	MHL	58.8	23.8	R	Mastoidectomy, Tympanoplasty
9	F	37	CHL	60	17.5	L	Malformation, Congenital, several ear surgeries; postoperative Epilepsy/Cavernoma Surgery, Levetiracetam therapy
10	F	50	MHL	77.5	13.8	R	Cholesteatoma; 2 × ear surgery TORP right, Tympanoplasty Implant right
11	F	45	MHL	41.3	18.8	L	Cholesteatoma
12	M	68	MHL	71.3	11.3	L	Radical Cavity both sides, BCI 601 on the contralateral right side 4 years before BCI 602 on the left side
13	M	45	MHL	73.8	12.5	R	Malformation
14	M	10	CHL	76.25	6.25	R	Malformation
15	M	9	CHL	22.5	2.5	L	Malformation
16	F	9	CHL	68.75	3.75	L	Malformation; EAC Atresia; Dysplasia grade III
17	F	6	CHL	58.75	8.75	R	Malformation; Atresia
18	F	12	MHL	56.25	13.75	R	Malformation; Atresia; several EAC-surgeries; Stenosis
19	M	5	MHL	63.75	17.5	R	Malformation
20	F	5.5	CHL	48.75	3.75	R	Malformation
21	F	5	CHL	60	12.5	R	Malformation
22	F	6	CHL	52.5	3.75	R	Malformation
23	M	9	CHL	52.5	0	L	Malformation
24	F	40	SSD	3.5	0.25	L	Acoustic Neuroma Surgery
25	F	55	SSD	16.25	15	L	Sudden deafness
26	M	63	SSD	16.25	16.25	L	Fractured cochlea
27	F	26	SSD	7.5	5	L	Congenital
28	F	41	SSD	17.5	12.5	L	Labyrinthitis
29	F	35	SSD	1.25	5	R	Meningitis
30	M	27	SSD	12.5	3.75	L	Congenital
31	F	9	SSD	2.5	-5	R	CMV—Cytomegalovirus
32	M	15	SSD	2.5	3.75	L	Congenital
33	M	69	SSD	15	13.75	L	Mastoidectomy

10 paediatric CHL/MHL patients were analysed [six female and four male; seven right-side and three left-side implants; average age of 8 years (range 5–12)] (see Table 1). The underlying aetiology for all paediatrics’ hearing loss were malformations, further specified as atresia in three cases. Two paediatrics suffered from MHL.

10 patients suffered from SSD, with severe to profound hearing loss on one side and normal hearing (NH) (see Table 1) on the other. Of the 10 SSD subjects, six were female and four male, with an average age of 38 years (range 9–69), two of which were under 18. Two were implanted on the right and eight on the left side. The underlying aetiology was either congenital, tumorous, infectious, or traumatic.

### Safety

#### Surgery

The average duration of surgery for the CHL/MHL group was 57 ± 19 min for adults and 57 ± 20 min for the paediatric group. The SSD group duration of surgery was shorter at 42 ± 3 min; the average for all patients was 52 ± 18 min (range 26–101 min).

The BC-FMT was placed in different regions of the temporal bone but mostly in the mastoid in area 3 (sinodural angle) and 4 (retrosigmoidal), as well as above the temporal line in areas 1 (above area 3) and 2 (above area 4) or combinations thereof (see Fig. 1 and Table 2). The incision usually ran from areas 3 to 1. On average, the incision spanned 5.5 ± 2.9 cm for the adult, 4.3 ± 1.3 cm for the paediatric and 4.8 ± 0.8 cm for the SSD groups. The average skin flap thickness was 4.5 mm for the adult, 4.6 mm for the paediatric and 4.7 mm for the SSD groups. Average cortical bone thickness was reported to be 4 mm for the adult, 2.7 mm for the paediatric and 4.3 mm for the SSD populations. The BCI 602 transition was bent horizontally upwards in 23 cases at an average angle of + 38° and downwards in 8 cases at an average angle of − 36°. Vertically, the BCI 602 transition was bent − 11° on average in 12 cases. The coil section was mostly placed in area B. This is the desired area as this locates the AP above the pinna and the microphones can pick up sound in an unblocked manner.

**Table 2:** Surgical evaluation. DNT = did not test; NA = not available

ID	Skin flap thickness [mm]	Incision area				Incision length [cm]	BC-FMT Distance to the ear canal [mm]	Cortical bone thickness [mm]	Dura exposed (and compressed)	Sinus exposed (and compressed)	FMT area				Coil area			Bent horizontal [°]	Bent vertical [°]	Times bent	Surgery time [hh:mm]
1	6	1		3		7	25	2	No	No			3				C	0	0	0	1:00
2	4			3		4	15	2	No	No			3			B		10	– 5	2	0:51
3	5			3		4	10	3	Yes	No				4		B		– 30	– 10	1	0:30
4	3.5			3		4.5	15	2	No	No	1					B		3	0	1	0:26
5	4			3		4	20	2	No	No	1					B		10	0	1	0:31
6	5	1		3		4.5	NA	4	No	No			3			B		90	0	1	1:05
7	6			3		3.5	NA	4	No	No			3			B		45	– 5	1	1:14
8	4	1	2	3	4	5	20	5	No	No	1		3			B		– 30	0	1	1:05
9	4	1		3		15	NA	5	Compressed	Yes		2				B		45	– 10	1	1:30
10	4	1		3		5	20	4.5	No	No				4		B		0	0	0	0:44
11	4.5	1		3		5	Unclear	2	Yes	No			3	4	A	B		60	– 10	2	1:16
12	3			3		5	40	4.5	Yes	No			3	4	A		C	– 20	0	1	1:10
13	4	1		3		5	10	6.2	Yes	No	1					B		30	NA	1	1:04
14	4	1		3		4	NA	5	No	No	1	2	3	4		B		30	0	1	0:39
15	4	1		3		5	20	4.3	Yes	No	1	2	3	4		B		30	0	1	1:41
16	4	1		3		4	NA	2.5	No	No			3	4		B		– 70	0	1	0:52
17	4			3		5	3	1.3	No	Compressed			3	4		B		10	0	1	1:00
18	5	1		3		7	3	2	No	No			3			B		60	0	1	0:32
19	5		2		4	2	30	5	No	No			3			B		40	– 10	1	0:45
20	5.5	1	2	3	4	3	5	4.4	Yes	Yes			3			B		45	0	1	1:01
21	5					3	21	4.5	No	NA			3	4		B		45	– 10	1	1:13
22	4.5	1		3		5	20	4	Yes	No	1					B		45	– 10	1	1:10
23	4.5	1		3		5	15	2.5	Compressed	Compressed	1		3					– 30	– 5	1	0:45
24	4	1		3		3.5	25	3.5	No	Yes	1	2	3	4		B		70	0	1	0:40
25	7	1		3		4	3.8	2	No	No			3			B		30	0	1	0:40
26	4			3		4	10	5	No	No			3	4		B		40	0	1	0:45
27	3			3		4	10	4	No	No			3			B		10	– 45	1	0:50
28	5	1		3		5.5	5	7.2	No	No			3			B		90	– 10	1	0:38
29	5.5	1		3		5	6	DNT	Yes	Yes			3			B		– 50	0	1	0:40
30	5	1		3		5.5	5	DNT	No	No			3			B		– 40	0	1	0:41
31	4	1		3		5	5	4	No	No			3			B		20	0	1	0:40
32	4	1		3		5	5	4	Compressed	Yes			3			B		20	0	1	0:40
33	5	1		3		6	10	4.5	Yes	No	1				A	B		– 20	– 5	1	0:46

No complications with the Surgical Screwdriver SD 2 were reported. No BCI 602 Lifts (1 mm) were used in any of the CHL/MHL patients, and the standard self-drilling screws were applied. In only one SSD patient (number 27 in Table 2), two Lifts were used and in two patients an emergency screw was used to “achieve better torque” and “better fixation”, respectively.

The dura was exposed 9 times (Adult *N* = 4; Paediatric *N* = 3; SSD *N* = 2). One of these patients reported as swelling around the coil section (see Adverse Events—ID 11). In 2 patients the dura was compressed, and the sinus exposed (Adults *N* = 1 and SSD *N* = 1). One of these suffered a bacterial infection of the skin flap (see AEs—ID 9). In one case the dura and the sinus were compressed (Paediatric *N* = 1). In one SSD patient the sinus was only exposed and in one paediatric case the sinus was compressed (see Table 2). The two ADEs reported were not related to the dura exposure or compression.

#### Adverse events

No intraoperative events were reported. For all enrolled ears 3 events related to the procedure or the device (Adverse Device Effects—ADE) were reported 3 months after surgery. All ADEs were anticipated and were classified as transient postoperative side effects. Two ADE were reported for the adult CHL/MHL and one ADE for the SSD population. One adult (ID 9) experienced a bacterial infection of the skin flap in the first month after surgery. This event was solved by antibiotic treatment and not wearing the AP for 14 days. This patient was pre-operated several times (see Table 1). Two months after surgery, one other adult patient (ID 11) reported swelling around the coil of the implant and pain when not wearing the AP that increased with extra pressure on the area. This event was solved with antibiotic treatment and not wearing the AP, as the patient had been wearing it for 15 h a day with a too strong magnet. After a period of discontinued AP usage, the magnet was switched to a weaker strength. One 9-year-old SSD patient experienced pain after surgery; changing the magnet strength from strength #2 to #1 solved the problem.

#### Pure tone thresholds

**Fig. 2:**
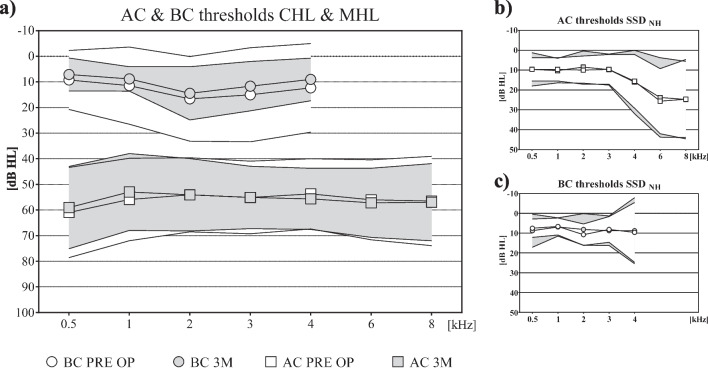
**a** AC and BC thresholds: CHL/MHL adults and paediatrics; **b** AC thresholds: SSD NH; **c** BC thresholds: SSD NH; square = mean AC; circle = mean BC; white = pre-operative; grey = 3-month post-operative; outer lines = standard deviations

Residual hearing by mean AC and BC thresholds (treated ear CHL/MHL and NH ear in SSD) was preserved 3 months after surgery. Results of pairwise comparisons confirmed that AC (Adult: *N* = 13, pre 56.3 ± 13.6 dB HL, 3 M 54.6 ± 13.5 dB HL; *t* = 1.377, *p* = 0.412; Paediatric: pre 56.0 ± 14.4 dB HL, 3 M 56.5 ± 11.3 dB HL; *z* = – 0.771, *p* = 0.44; SSD: pre 9.5 ± 6.7 dB HL, 3 M 9.9 ± 7.6 dB HL; *t* = – 0.316, *p* = 0.759) and BC (Adult: *N* = 12, pre 11.9 ± 6 dB HL, 3 M 11.5 ± 5.6 dB HL; *t* = 0.343, *p* = 0.356; Paediatric: pre 7.3 ± 5.7 dB HL, 3 M 7.1 ± 4.9 dB HL; *t* = 0.105, *p* = 0.788; SSD: pre 6.9 ± 6.1 dB HL, 3 M 9.9 ± 7.6 dB HL; *t* = − 0.779, *p* = 0.456) PTA_4_ thresholds remained stable over time from preoperative to 3-month post-operative. No clinically significant decrease (> 10 dB HL) at 3-month post-operative in mean AC and BC PTA_4_ and mean single frequency thresholds (see Fig. 2) was observed.

### Performance

#### Wearing time

Mean AP wearing times in hours per day for the adult CHL/MHL group were 13 h [h] 12 min [min] ± 4 h 54 min, 10 h 42 min ± 3 h for the paediatric CHL/MHL group and 6 h 20 min ± 3 h 13 min for the SSD group.

#### Sound field thresholds

**Fig. 3:**
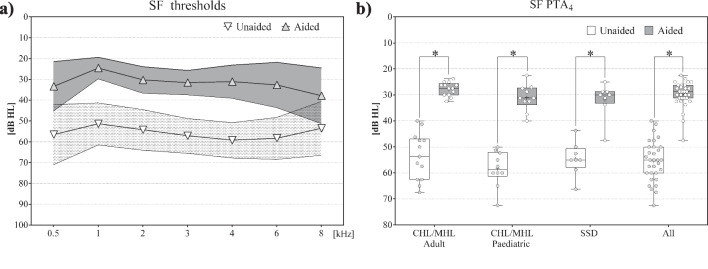
**a** SF thresholds: all subjects; inverted triangle and white = mean unaided; triangle and grey = mean aided; outer lines = standard deviations. **b** SF PTA 4 thresholds: Box Plots, median = horizontal lines, +  = mean, circles = individual values; * = significance

The endpoint was reached with a mean improvement of ≥ 10 dB HL. Mean SF PTA_4_ thresholds improved significantly for all subjects (All: *N* = 31, *z* = – 4.862, *p* < 0.001*; Adult: *N* = 13, *t* = 10.172, *p* < 0.001*; Paediatric: *N* = 10, *t* = 18.983, *p* < 0.001*; SSD: *N* = 8, *z* = – 2.536, *p* < 0.011*) from unaided 55.4 ± 7.7 (Adult: 53.7 ± 9.0 dB HL; Paediatric: 58.4 ± 6.9 dB HL; SSD: 54.5 ± 6.1 dB HL) to aided 29.9 ± 4.9 dB HL (Adult: 27.7 ± 2.6 dB HL; Paediatric: 31.1 ± 5.1 dB HL; SSD: 31.9 ± 6.8 dB HL), with a PTA_4_ functional gain (FG) of 25.5 dB (Adult: 26.0 dB; Paediatric: 27.3 dB; SSD: 22.7 dB) (see Fig. 3 and Table 3).

**Table 3:** SF thresholds [dB HL]. STD = standard deviation

Average		56.6	51.4	54.3	57.2	59.3	58.4	53.6	55.4	33.4	24.6	30.3	31.6	31.1	32.7	37.9	29.9
STD		14.2	9.9	9.5	8.1	8.3	9.9	12.7	7.7	11.7	5.1	6.2	5.7	7.8	10.7	13.1	4.9
*N*		31	31	31	31	31	31	31	31	31	31	31	31	31	31	29	31
HL type	Adult pediatric	Unaided								Aided							
		0.5	1	2	3	4	6	8	PTA4	0.5	1	2	3	4	6	8	PTA4
CHL/MHL	Adult	60	55	60	60	55	45	35	57.5	30	25	35	40	30	25	30	30
CHL/MHL	Adult	20	25	55	65	60	55	50	40.0	25	15	25	35	35	25	35	25
CHL/MHL	Adult	50	50	40	50	50	55	45	47.5	25	25	30	20	25	25	30	26.25
CHL/MHL	Adult	70	60	60	55	70	50	40	65.0	20	20	25	30	30	35	40	23.75
CHL/MHL	Adult	65	45	50	55	65	65	80	56.3	30	15	20	30	40	40	50	26.25
CHL/MHL	Adult	55	50	40	45	45	55	55	47.5	30	30	30	30	35	30	30	31.25
CHL/MHL	Adult	50	45	45	40	45	45	40	46.3	35	20	25	30	30	25	35	27.5
CHL/MHL	Adult	30	35	55	65	80	75	70	50.0	20	30	35	45	45	45	50	32.5
CHL/MHL	Adult	55	50	55	55	55	60	45	53.8	30	25	25	25	20	25	25	25
CHL/MHL	Adult	60	55	65	65	70	55	45	62.5	20	25	35	35	30	30	45	27.5
CHL/MHL	Adult	45	40	30	40	50	45	55	41.3	35	30	20	30	20	25	30	26.25
CHL/MHL	Adult	90	60	55	70	65	80	70	67.5	45	25	30	25	20	25	30	30
CHL/MHL	Adult	70	60	65	65	55	60	40	62.5	35	25	30	30	25	15	25	28.75
CHL/MHL	Pediatric	75	70	70	75	75	70	70	72.5	50	30	40	40	40	45	50	40
CHL/MHL	Pediatric	45	48	58	62	58	60	46	52.3	25	23	30	30	30	30		27
CHL/MHL	Pediatric	70	65	55	50	50	55	50	60.0	35	35	35	25	25	30	30	32.5
CHL/MHL	Pediatric	75	60	65	60	60	50	45	65.0	40	25	30	25	20	15		28.75
CHL/MHL	Pediatric	55	60	65	60	60	60	50	60.0	45	35	40	35	30	35	45	37.5
CHL/MHL	Pediatric	65	55	60	50	60	50	45	60.0	35	20	35	35	35	25	20	31.25
CHL/MHL	Pediatric	50	45	65	65	70	60	60	57.5	35	30	30	30	30	30	30	31.25
CHL/MHL	Pediatric	45	50	65	55	60	50	55	55.0	20	25	45	35	40	40	40	32.5
CHL/MHL	Pediatric	60	40	45	50	55	40	30	50.0	30	20	30	30	30	35	25	27.5
CHL/MHL	Pediatric	50	50	50	55	55	45	45	51.3	20	15	35	25	20	25	25	22.5
SSD	Adult	55	40	45	65	60	70	55	50.0	20	20	20	40	40	55	45	25
SSD	Adult	45	50	60	60	65	70	60	55.0	25	25	35	35	40	40	25	31.25
SSD	Adult	75	60	65	65	65	65	75	66.3	75	30	35	35	50	45	65	47.5
SSD	Adult	70	55	40	60	55	65	65	55.0	45	25	20	25	30	30	45	30
SSD	Adult	60	70	55	55	50	55	45	58.8	50	20	25	40	40	35	50	33.75
SSD	Pediatric	40	40	45	50	50	75	80	43.8	40	25	25	25	30	60	70	30
SSD	Pediatric	45	55	50	50	60	65	60	52.5	30	25	35	35	25	50	60	28.75
SSD	Adult	55	50	50	55	65	60	55	55.0	35	25	30	30	25	20	20	28.75

#### Speech intelligibility WRS (CHL/MHL)

**Fig. 4:**
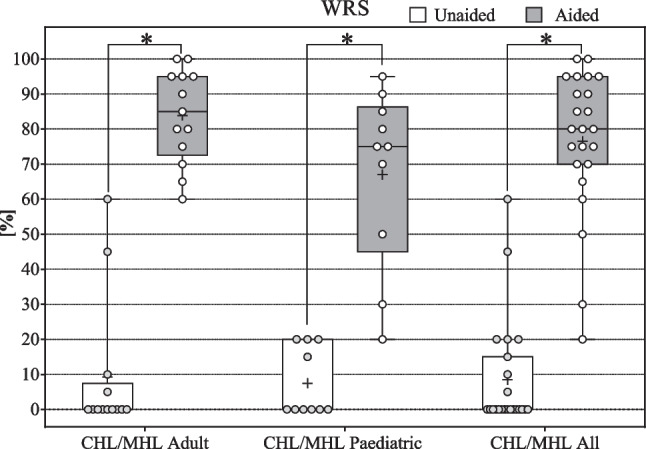
WRS CHL/MHL: Box Plots, median = horizontal lines, +  = mean, circle = individual values; * = significance

The endpoint was reached with a mean improvement of ≥ 15% SPL. Mean WRS improved significantly for all CHL/MHL subjects (All: *N* = 23, *z* = – 4.202; *p* < 0.001*; Adult: *N* = 13, *z* = – 3.183, *p* < 0.001*; Paediatric: *N* = 10, *z* = – 2.807, *p* < 0.001*) from unaided 8.48 ± 15.8% (Adult: 9.2 ± 19.7%; Paediatric: 7.5 ± 9.8%) to aided 76.5% (Adult: 83.9 ± 13.4%; Paediatric: 67.0 ± 25.41%) by 68.0% (Adult: 74.6%; Paediatric: 59.5%). The endpoint was reached with a mean improvement of > 15% (see Fig. 4).

#### Speech intelligibility SRT in quiet (CHL/MHL)

**Fig. 5:**
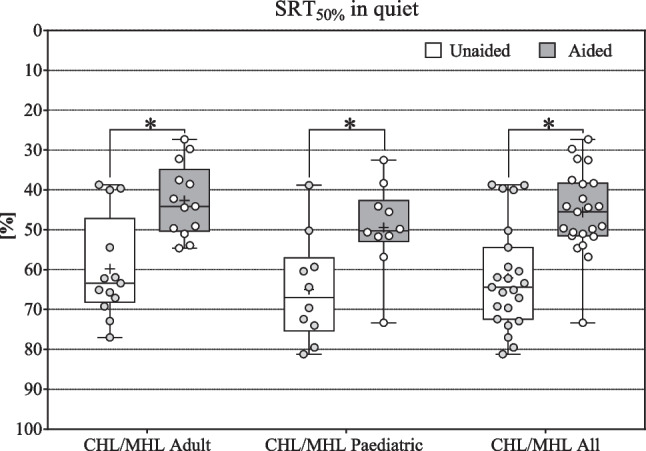
SRT in quiet CHL/MHL: Box Plots, median = horizontal lines, +  = mean, circle = individual values; * = significance

The endpoint was reached with a mean improvement of ≥ 15 dB SPL. Mean SRT in quiet improved significantly for all CHL/MHL subjects (All: *N* = 23, *z* = – 4.136; *p* < 0.001*; Adult: *N* = 13, *z* = – 3.04, *p* < 0.002*; Paediatric: *N* = 10, *t* = – 6.567, *p* < 0.001*) from unaided 62.0 ± 12.9 dB SPL (Adult: 59.0 ± 12.8 dB SPL; Paediatric: 65.0 ± 13.3 dB SPL) to aided 45.6 ± 10.3 dB SPL (Adult: 42.6 ± 9.1 dB SPL; Paediatric: 49.4 ± 11 dB SPL) by 16.5 dB (Adult: 17.2 dB; Paediatric: 15.6 dB) The endpoint was reached with a mean improvement of > 15 dB SPL (see Fig. 5).

#### Speech intelligibility SRT in noise

Mean SRT in noise improved significantly for all CHL/MHL subjects in S0°N0° (All: *N* = 23, *z* = – 4.198; *p* < 0.001*; Adult: *N* = 13, *z* = – 3.181, *p* < 0.001*; Paediatric: *N* = 10, *z* = – 2.803, *p* < 0.001*), from unaided 3.55 ± 7.5 (Adult: 2.4 ± 4.6 dB SNR; Paediatric: 5.05 ± 10.2 dB SNR) to aided − 3.47 ± 3.4 dB SNR (Adult: − 4.4 ± 1.8 dB SNR; Paediatric: − 2.28 ± 4.6 dB SNR) by − 7.02 dB (Adult: − 6.8 dB; Paediatric: − 7.33 dB). The endpoint was reached with a mean improvement of > − 1 dB (see Fig. 5a).

In the SSD population, with speech applied from the SSD ear and noise from the normal-hearing ear (S_SSD_N_NH_, *N* = 10), the mean SRT in noise improved significantly (*t* = 5.365, *p* < 0.001*), by − 3.51 dB SNR from + 2.47 to − 1.04 dB SNR. With speech applied from the front and noise from the normal-hearing ear (S0°N_NH_, *N* = 10), a tendency for a statistically significant improvement (*t* = 2.250, *p* = 0.051) of –0.8 dB from + 0.15 to − 0.65 dB SNR was reported. The endpoint was reached with a mean improvement of ≥ − 1 dB S_SSD_N_NH,_ but not in S0°N_NH_ (see Fig. 5b).

## Discussion

### Safety

#### Surgery

The average surgery for the BCI 602 lasted 52 ± 18 min (range 26–101 min) for all patients. A shorter duration of surgery further underlines the safety and ease of implantation of the BCI 602. Average surgical durations of 55 ± 23 min and 53.7 min (range 30–158 min) are reported for the BCI 601 [12] and for the OSIA2 [18–20], respectively.

An advantage of the BCI 602 as compared to other transcutaneous BCIs is the possibility to bend the transition to achieve an optimal placement of the AP. This feature was widely used with the BCI 602 (horizontal *N* = 31; vertical *N* = 12). The BCI 602 was mostly placed in the mastoid (Area 3, sinodural angle; Area 3 + 4, retrosigmoidal) or above the temporal line (Area 1 or Area 1 + 2); only one patient was implanted in Area 2 (see Table 2). Implantation in Area 1 and 2 is described for the BCI 601 [21–23] in patients with previous surgeries or other anatomical restrictions. Regardless of the placement of the implant, the audiological endpoints were reached.

Der et al. and Carnevale et al. report on successful placement of the BCI 601 above the temporal line with similar audiological outcomes to the outcomes reported in this study [23]. The latter report a duration of surgery of 47 min using a standard otological drill and 28 min with the Neuro Drill for this approach.

Interestingly, no BCI 602 Lifts were used in the paediatric as well as in the adult groups, even though the average cortical bone thickness was thinner at 3.6 mm and 3.7 mm, respectively, compared to the 4.3 mm of the SSD population in which two Lifts were used in one patient. This SSD patient showed a significant improvement of − 2.4 dB SNR in setup one (S_SSD_N_NH_). This improvement is in the range (− 1.31 to − 5.5 dB) reported for the BCI 601. For comparison, the implantation depth of the BC-FMT is 4.5 mm without Lifts and 3.5 mm with Lifts, and the total bone thickness is larger than the cortical bone thickness [24].

Yang et al. focused on compressions and use of Lifts in Bonebridge implantation [24]. They analysed the mean anteroposterior mastoid bone thickness in 110 bilateral congenital microtia BCI 601 patients with a mean age of 11.7 ± 5.2 years. The anteroposterior mastoid bone thickness was measured from the external auditory canal to the sigmoid sinus. They found statistically different (*p* < 0.001) anteroposterior mastoid bone thickness in the non-compression group of 16.2 ± 2.3 mm (*N* = 67) and in the compression group of 13.1 ± 2.9 mm (*N* = 43; dura: 18 patients, sinus: 14 patients, both: 11 patients), without any differences in performance. 42 patients were implanted with Lifts (5 × 1 mm, 26 × 2 mm and 11 × 3 mm Lift). In 26 patients the Lifts prevented compression, while in 16 patients, the Lifts could not prevent compression. As mentioned before, compression of either the dura or sigmoid sinus or use of Lifts had no effect on hearing outcomes. With respect to audiological outcomes, no differences using Lifts, or no Lifts were also reported by Brkic et al. [25].

Furthermore, no significant differences were found in a study measuring sound transmitted to the cochlea in cadaveric temporal bones in respect to screw type, Lift thickness, or implant location [26].

Exposure and compression did not lead to any complications in this study. The different implant placements used underline the fact that implantation in the temporal bone allows for treatment of normal, pre-operated and malformed anatomies.

Loader et al. [27] compared the audiological and surgical outcomes in mastoidal and retrosigmoidal placement of the BCI 601 and found no statistically significant differences. Kulasegarah et al. showed that children with atresia and microtia and even ear reconstructions have good outcomes with the BCI 601 but stated that in some children with small mastoids a BCI 601 implantation is not possible [28].

Auricular reconstruction and simultaneous BCI 601 implantation in uni- and bilateral microtia patients has been well-described by Chan et al. [29] and Wang et al. [30], underlining the fact that Bonebridge treatment does not prevent aesthetic treatment. Similarly, the simultaneous implantation of the Bonebridge hearing implant system together with anchors for individual auricular prosthesis is an adequate option for simultaneous cosmetic and audiological rehabilitation [31].

Wenzel et al. showed by three-dimensional reconstruction of temporal bones from computed tomography and virtually implanting the BCI 602 and BCI 601 in 151 mastoids, that the newer BCI 602 transducer can more likely than its predecessor be completely accommodated in the mastoid without using Lifts (100% in people aged 12 years and older and 75% of 3–5 years) [6]. Using the Lifts, the BCI 602 could be virtually implanted in 81% or more of cases aged three and above. This study showed that the reduced drilling depth required for the BCI 602 may also allow for placement in patients with thin mastoid bones or malformations. Since Wenzel et al. focused on the mastoid area and not on the whole temporal bone, placement options for the BCI 602 should be even more variable.

#### Adverse events

Safety of the device was confirmed by a lack of significant change in bone and air conduction thresholds in the normal hearing ear (see Fig. 2). Safety of the BCI 602 treatment was further established, as only transient postoperative side effects occurred, all of which could be solved by antibiotics, not wearing the AP, for a short time, changing the magnet strength or combinations thereof. Regarding dura or sinus compression (see Table 2), no intraoperative side effects were reported.

### Performance

#### Wearing time

The BCI 602 device was used for ~ 11 h a day in the paediatric CHL/MHL population, which is in line with wearing times for the BCI 601 (~ 12 h, [12]), but less than reported for the adult population (~ 13 h). The wearing time of ~ 6 h is shorter for the SSD population. This may be attributed to the normal-hearing ear in SSD patients. Wearing times of ~ 8 h for the BCI 601 in SSD patients are reported 2 years after surgery [11].

#### Sound field thresholds

Hearing with the BCI 602 in SF PTA_4_ showed significant improvements from 55.40 dB HL unaided to 29.86 dB HL with the Bonebridge. The primary endpoint of 10 dB improvement was reached, with mean functional gains (FG) of 25.53 dB for all subjects and 26 dB for the adult, 27.27 dB for the paediatric and 22.66 dB for the SSD populations (see Fig. 3). These findings are in agreement with published literature on the BCI 601, with fifteen studies reporting FGs ranging from 23 to 40 dB [4, 9, 10, 32–44], thirteen studies reporting FGs for paediatric patients ranging from 23.1 to 39.8 dB [10, 22, 24, 28, 44–52] and eleven studies in 103 SSD subjects reporting FGs with a range from 17 to 71.5 dB [38, 39, 41, 53–60].

#### Speech intelligibility

**Fig. 6:**
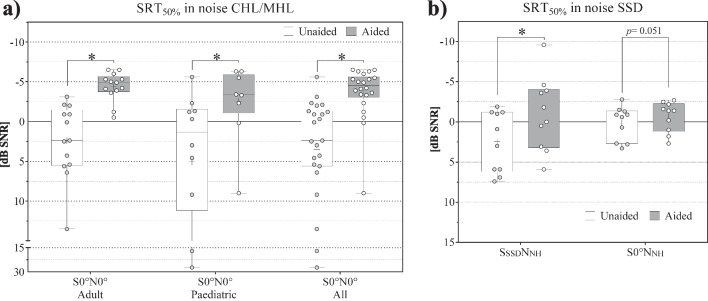
SRT in noise **a** CHL/MHL in S0°N0°; **b** SSD in S_SSD_N_NH_ and in S0°N_NH_; Box Plots: median = horizontal lines, +  = mean, circle = individual values; * = significance

The primary endpoint of 15% mean improvement in WRS was reached with 68.04% (see Fig. 4) for all CHL/MHL patients and corresponds to the improvements of 40–95% reported for the BCI 601 [9, 10, 32–41, 43, 44, 61]. This improvement is underlined by significant improvements of 16.47 dB in SRT in quiet (see Fig. 5) and − 7.02 dB in SRT in noise (Fig. 6a) for the CHL/MHL population.

In addition to the improvement of hearing thresholds, the main advantage of a Bonebridge for an SSD patient is for speech understanding in noise. The biggest effect is expected when noise is presented at the normal hearing side and the signal is coming from the implanted side (S_SSD_N_NH_) or the front (S0°N_NH_). In S_SSD_N_NH_ the improvement of − 3.51 dB was significant (see Fig. 6b).

This is in line with five other publications on the BCI 601 in this setup (S_SSD_N_NH_) that reported an average improvement of − 3.3 dB (range − 1.31 to − 5.5 dB) [35, 38, 40, 61, 62]. In S0°N_NH_, the improvement of − 0.8 dB has a tendency to significance (see Fig. 6b). This outcome is comparable to the average improvements reported for the BCI 601 in a similar setup (S_SSD_N0°), with a range of + 0.3 to − 2.5 dB [11, 40, 53, 61–64].

## Conclusions

The latest generation implant of the Bonebridge system, the BCI 602—the first active transcutaneous hearing implant—significantly improves hearing thresholds and speech understanding in patients suffering from CHL/MHL and in patients treated for SSD on short-term follow-up. The new geometry of the BCI 602 allows for more placement options that may be advantageous for use in patients with thin bones, malformations, or pre-operated ears. Only three minor device-related events were reported, demonstrating an unaltered safety profile.

## Data Availability

The datasets used and/or analysed during the current study are available from the corresponding author on reasonable request.
